# Decrementally cost-effective health technologies in non-inferiority studies: A systematic review

**DOI:** 10.3389/fphar.2022.1025326

**Published:** 2022-12-05

**Authors:** Meryl Darlington, Raffaele Scarica, Xyomara Chavez-Pacheco, Laeticia Blamplain Segar, Isabelle Durand-Zaleski

**Affiliations:** ^1^ Assistance Publique-Hôpitaux de Paris (AP-HP), Hôpital Hôtel Dieu, Clinical Research Unit Eco Ile de France, Paris, France; ^2^ Université de Paris Est Creteil INSERM UMRS, Paris, France

**Keywords:** systematic review, cost, economics, non-inferiority, health technology assessment

## Abstract

**Background:** HTA guidance has generally been driven by situations where innovative and usually more expensive technologies are compared to the prevailing standards of care. Cheaper and less efficacious interventions have received scarce attention, although strategies with minimal individual efficacy losses might produce collective health gains when savings are redistributed.

**Purpose:** This systematic review of health economic evaluations identified interventions that are both cost and outcome reducing to procure a list of candidate decrementally cost-effective technologies.

**Data Sources:** English language searches were performed in PubMed, EMBASE and ClinicalTrials.gov covering 2005 to September 2021.

**Study Selection:** Full economic evaluations reporting in English decrementally cost-effective health technologies based on RCT data, modelling or mixed methods.

**Data Synthesis:** After filtering 4,975 studies found through the systematic database search, 107 decrementally cost-effective health technologies (HTs) were identified. Nearly a third were services (n = 29) and similarly for drugs (n = 31). For over half of the studies (n = 54) health outcomes were measured in QALYs and the cost-utility ratios varied from €140 to €5 million saved per QALY lost, albeit with time horizons varying from 4 days of follow-up to lifetime extrapolations. Less than a quarter of the studies were carried out from the societal perspective.

**Limitations:** Despite including ClinicalTrials.gov as data source, unpublished studies may have been missed.

**Conclusions:** Our results show a growth in recent years in the number of economic publications demonstrating decrementally cost-effective HTs. Economic tools are needed to facilitate the adoption of such HTs by policy-makers at the national level to maximise health outcomes at the population level.

**Systematic Review Registration:** https://www.crd.york.ac.uk/prospero/display_record.php?RecordID=95504, identifier CRD42018095504.

## Introduction

Since the 1970s Health Technology Assessments (HTAs) have been increasingly used to evaluate the efficacy and costs of Health Technologies (HTs). Against a background of increasing demands on limited resources, HTAs have a growing impact on health policy. The typical situations met in HTA consist of incremental innovations that are characterised by cost increases and efficacy enhancements compared to usual standards of care. These innovations belong to the north-east quadrant of the cost-effectiveness (C-E) plane (Black, 1990). The north-east quadrant implies trade-offs on how much society is willing to pay for the extra efficacy, the theoretical and empirical foundations of priority setting, pricing and reimbursement decisions nearly always relate to this quadrant. The south-west quadrant (lower cost/lower efficacy) has been given even less consideration; a review of published C-E analyses reported that only 2% of C-E studies were for interventions associated with lower cost and lower efficacy (Nelson et al., 2009). In settings where resources are limited, the adoption of cost-reducing technologies may lead to budget reallocation in order to improve health outcomes in other domains even if they lead to slightly worse individual outcomes in a specific disease or patient subgroup. Nonetheless, in Europe the development and diffusion of better medical interventions are more common, given that clinical research stakeholders are mostly encouraged to investigate the improvement of care quality, or at least to demonstrate equal care quality (Kent et al., 2004).

However, in the last decade, non-inferiority trials have gained attention among health stakeholders. In these trials, an alternative treatment has an efficacy similar to, or at least not much worse than, the standard treatment, with possible advantages regarding safety, convenience, better compliance, or cost reduction. A search of the Cochrane Controlled Trials Register for two periods of 10 years (1999–2009 and 2009–2019) demonstrated that the total number of trials registered worldwide increased threefold (from 0.3 million to over one million) whilst the number of non-inferiority or equivalence trials increased fourfold (from 6 K to 29 K). Currently, there is no guidance on decision-making for decrementally cost-effective (d-CE) interventions (health technologies associated with a cost and efficacy reduction profile that is deemed acceptable) and the reticence in accepting a small loss in quality-adjusted life years (QALYs) has not been accommodated in routine reimbursement decisions. The definition of a non-inferiority margin is based on both statistical reasoning and clinical judgment, under the assumption that the difference (decrease) in effect will not be harmful to patients. The concept of applying a non-inferiority margin to economic evaluations has been explored for model-based studies and requires the intervention to be cost-saving, non-inferior for the clinical outcome and also non-inferior for the quality of life dimension as measured by QALYs. However it is unusual to estimate non-inferiority margins for QALYs (Xie et al., 2019). Non-inferiority studies provide good material for economic evaluations which study the joint distribution of costs and outcome and represent uncertainty on the cost-effectiveness plane or through the use of the net-benefit statistic (Briggs and O’Brien, 2001). In some cases, the trade-offs associated with implementing d-CE strategies have been measured, yet no policy decisions have been systematically implemented. (Dowie et al., 2015).

The objective of this systematic review was to identify d-CE studies published recently in order to inform researchers and decision-makers about the d-CE technologies currently available.

## Materials and methods

This review was conducted in accordance with the five-step approach for systematic review of economic evaluations published in the “Expert Review of Pharmacoeconomics and Outcomes Research” journal (Thielen et al., 2016) (van Mastrigt et al., 2016) (Wijnen et al., 2016). The protocol was published on PROSPERO (Registration number: CRD42018095504) and was reported according to the Preferred Reporting Items for Systematic Reviews and Meta-Analyses Protocols (PRISMA-P) guidelines (Moher et al., 2009).

### Data sources and searches

Systematic electronic searches were conducted using PubMed, EMBASE and the Clinical Trials registry (https://clinicaltrials.gov/). Other databases were investigated with non-systematic searches such as Tufts, EuroCT, EBSCOhost, CRD York and ISRCTN as well as grey literature, published between 1st January 2005 and 4th October 2021. Manual searches were carried out using a snowballing technique and investigating citations found in pertinent articles. Full search strategies are provided in the Supplementary Appendix S1.

### Study selection

The inclusion criteria, that studies should demonstrate decremental C-E, would normally require definition of a threshold related to the willingness to accept (WTA) a loss in QALY for monetary gain. However, the efficacy in C-E studies can be measured in natural units (e.g., mmHg for blood pressure, HbA1c for diabetes) or in health utilities (QALY, Disability-adjusted life years, or other). Given that our review covers multiple countries having different criteria for evaluating C-E and that we included studies with efficacy measured in natural units, we did not use a threshold to determine inclusion or exclusion of a study. When the decremental C-E ratio (d-CER) was calculated and a C-E plane used to show the uncertainty around these results, we were able to identify that the cloud of points fell at least 50% in the south-west quadrant. Where this information was not available in the article, we checked the confidence intervals of the disaggregated data (costs and efficacy) to estimate that a cloud would almost certainly be at least 50% in the south-west quadrant.

**FIGURE 1:**
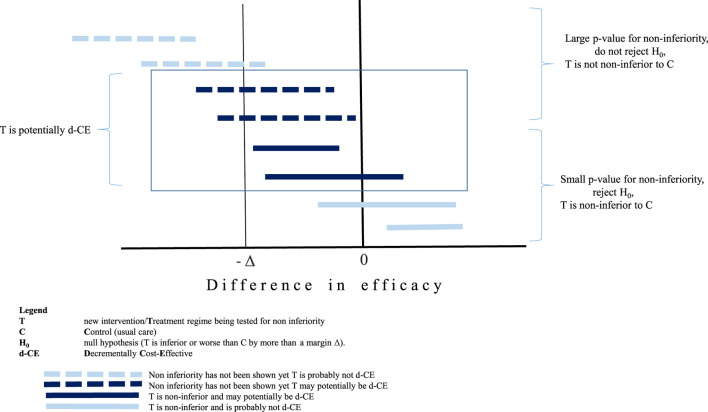
Non-inferiority studies and decremental cost-effectiveness.

Whilst this review focussed on technologies with a very strong economic rationale for implementation balanced by a weak medical rational, such as the non-inferior medical efficacy, when a health technology is found to be non-inferior to the comparator, the HT is not necessarily decrementally cost-effective as shown in Figure 1. The bottom four horizontal lines represent non-inferior technologies compared to usual care. Even in the event that the economic evaluation demonstrates large potential cost savings, two of these four (the two closest to the *x* axis) would not result in a d-CER since these technologies are actually superior to usual care. The top four horizontal lines results have not been shown to be non-inferior, yet there is a possibility that a C-E ratio for two of the examples shown could be of interest given that the point value is within the non-inferiority margin and the confidence intervals are right skewed from the margin value (as shown by Δ). Whilst from a clinical point of view, the classic rules of inference based on using the *p*-value to demonstrate significance of (in this case) non-inferiority are still applied, these are arbitrary rules and not relevant to the decisions which are informed by health economic evaluations (Claxton, 1999).

The search was conducted according to the following inclusion criteria: 1) the interventions were applied to human subjects; 2) the interventions were evaluated in a full economic evaluation as defined in Drummond et al. (Drummond and Jefferson, 1996) thus comparing at least two HTs with assessment of both costs and outcomes; 3) the interventions were evaluated in countries defined as an upper-middle-income or high-income economy according to the World Bank’s 2018 country classification by income level; 4) the interventions were traditional HTs according to the WHO definition: “the application of organized knowledge and skills in the form of medicines, medical devices, vaccines, procedures and systems developed to solve a health problem and improve quality of life”; 5) the interventions should be d-CE compared to the standard of care; 6) studies should be written in English.

**FIGURE 2:**
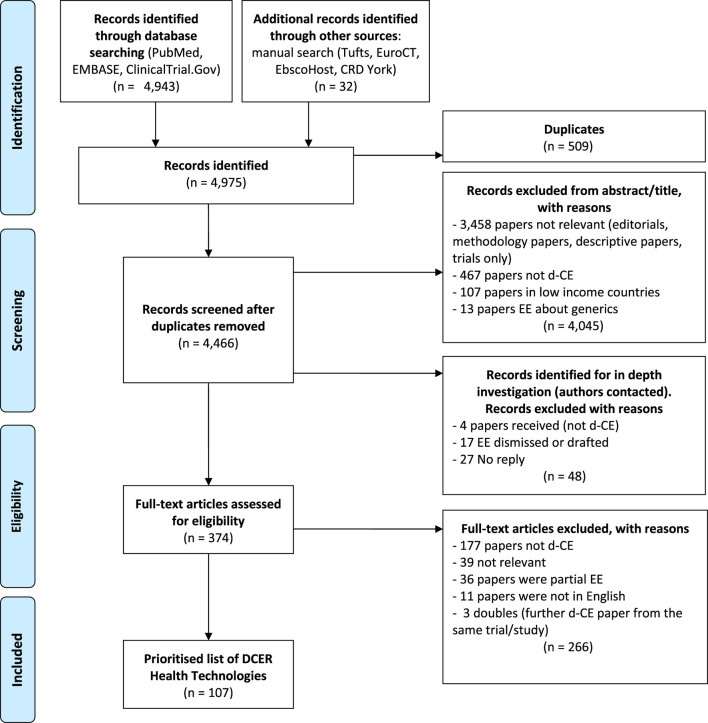
PRISMA flowchart of study selection.

Publications reporting on methodological issues, discussion articles, partial economic evaluations, HT including a generic component, comment letters and editorials were excluded. We excluded duplicates found in more than one database. The reasons for exclusion for each study were reported on a PRISMA flowchart (Figure 2). Studies comparing generic drugs to the commercial variety were excluded from the review. Biosimilar products, that are not identical to the original branded biologic and that must have their own clinical data and pharmacovigilance, were included. We carried out a systematic search of trials as well as protocols characterised as equivalence or non-inferiority results and for which an economic analysis was planned.

### Data extraction and quality assessment

The results of the search strategy in PubMed, Embase and ClincialTrials.gov were exported and managed in Excel files and Rayyan QCRI (https://rayyan.qcri.org/). Study selection was based on the inclusion and exclusion criteria and was carried out in double. Two reviewers (XC and RS) independently screened titles and abstracts using the inclusion criteria. Secondly, the full-text version was screened in double by three reviewers (XC, LBS, RS) and a final decision made with respect to the inclusion/exclusion criteria. Any disagreement or conflicting views between the reviewers over the eligibility of specific economic evaluations was resolved by discussion or the final judgment of a fourth reviewer (MD). Both stages of the selection process were piloted and if necessary modified. Studies found through trial registry records or published protocols were considered for in-depth investigations when the clinical non-inferior or equivalence results were published and an economic evaluation was planned for these trials. Internet searches were conducted to ascertain if any economic results had been published and in case of inconclusive findings, investigators were contacted to determine if an economic evaluation had been carried out or why the economic results had not been diffused.

We reviewed four checklists for quality assessment: Drummond Checklist (Drummond and Jefferson, 1996), Philips checklist (Philips et al., 2006), CHEERS checklist (Husereau et al., 2013) and the CHEC list (Evers et al., 2005), one for bias (Adarkwah et al., 2016) and three for transferability (Drummond et al., 2009) (Wijnen et al., 2016) (Welte et al., 2004). The three components of quality, bias and transferability had a certain amount of overlap in the questions and we collated the questions and eliminated redundancy from the different sources to create a reduced list shown in Supplementary Materials Appendix 3. The final list used for screening full text articles had 22 questions. In order to calculate a quality score, a value of 1, 0.5, 0 or not applicable (NA) was given to each question. A score of 1 indicated that the reviewer considered that the article fully satisfied the question. A score of 0 indicated that the paper did not satisfy the criteria at all. The score of 0.5 was awarded when it seems that some attempt had been made to address the question but that it was not completely adequate. The option NA was selected in cases where it was not appropriate to answer the question. For example, if the time horizon was 1 year or less then discounting would not be carried out and NA was coded for this question (item 10 on the checklist). The overall score of the paper was the sum of the score for each question divided by the number of applicable questions.

### Data synthesis and analysis

Publication information, study characteristics and findings from the included studies, related to the research question, were gathered in a database form using Excel. The data extraction list from Wijnen et al. (Wijnen et al., 2016) was used as a basis and other items were included that are directly related to non-inferiority or equivalence trials such as study analysis approach of intention to treat *versus* per protocol. When the d-CER was not reported, it was calculated where possible by dividing the differential cost and the differential effect (QALY, Life Years, other) found in the text. Given the different locations, years of study and country-specific elements such as different currencies, the costs were converted into a common currency and price year using the CCEMG—EPPI-Centre Cost Converter as recommended in the five step methodology, which enable us to convert and adjust the d-CER of each article to 2022 euros (€) (https://eppi.ioe.ac.uk/costconversion/).

## Results

In total, 4,975 records were found from PubMed, EMBASE, ClinicalTrials.gov and the manual searches. The latter included the results retrieved by using the snowballing technique among the rest of databases such as Tufts, EuroCT, EBSCOhost, CRD York and ISRCTN. After filtering studies according to the inclusion criteria, we found 107 published d-CE economic evaluations, representing 107 days-CE HTs as shown in the PRISMA flowchart (Figure 2). The scope of the articles varied considerably and not all of the published economic evaluations reported the d-CER; when it was not possible to be calculated, the information was shown in disaggregated form. The full list of included studies with key characteristics is available in the supplementary material (appendix 2). Nearly 30% of the 107 HTs were services (n = 29) and similarly for drugs (n = 31). These papers, that were predominantly about cancer, cardiovascular diseases, musculoskeletal disorders and respiratory diseases, were almost equally split between new/alternative technologies (n = 54) and strategies that were using the same technology (n = 53) such as drug tapering studies. Over half of the studies were publicly funded (n = 67) and were primarily carried out in the USA (n = 28) and the UK (n = 23) which reflects the importance and quantity of economic studies in general carried out in those countries. Over half of the economic evaluations were conducted alongside randomised control trials (n = 65).

For the studies where it was possible to calculate the decremental cost-utility ratio, it ranged from €151 to €5,044,460 saved per QALY lost. Table 1 shows the key characteristics of interventions with a point estimate of the cost utility ratio above €100,000 saved per QALY lost. The time horizons varied from 4 days to lifetime extrapolations in the case of modelling studies. For over half of the studies (n = 54) health outcomes were expressed in QALYs, in other cases effectiveness was measured in natural units or functional scales.

**TABLE 1:** Key characteristics of studies with a point estimate of the cost utility ratio greater than €100,000/Qaly lost.

Author	Year/country		Disease	Intervention	Type	Effects and time horizon		DCER 2022 €
Bansback et al. (2017)	2017	US	Rheumatoid arthritis	Triple Therapy	RCT	-0.016 QALY	48 weeks	€ 897 558/QALY Lost
Blondon et al. (2020)	2020	US	Pulmonary embolism	Age-adjusted cutoff	Decision Model	- 0.0001 QALYs	Lifetime	€188 361/QALYs lost
Brown et al. (2018)	2018	UK	Rheumatoid arthritis	Etanercept/Adalimumab	Mixed	-0.02 QALY	2 years	€ 242 916/QALY Lost
Clark et al. (2015)	2015	UK	Abnormal uterine bleeding	Outpatient	RCT	-0.006 QALYs	1 year	€ 206 490/QALY Lost
Corral et al. (2017)	2017	Spain	Obstructive sleep apnoea	HRP Home respiratory polygraphy	RCT	-0.004 QALYs	6 months	€ 144 555/QALY Lost
Cram et al. (2006)	2006	US	Cardiac Arrest	Automated external defibrillators (AEDs)	Decision Model	-0.85 QALYs	Lifetime	€ 125 018/QALY Lost
Cross et al. (2010)	2010	UK	COPD	Manual chest physiotherapy	RCT	- 0.001 QALYs	6 months	€605 380/QALY lost
Dakin et al. (2014)	2014	UK	Neovascular age-related macular degeneration (nAMD)	Continuous Bevacizumab	RCT	-0.004 QALY	2 years	€ 5 185 700/QALY Lost
Dickson et al. (2011)	2011	UK	Lung Cancer	Erlotinib	Mixed	-0.1007 QALYs	Life-time	€123 809/QALY lost
Ferket et al. (2017)	2017	US	Osteoarthritis	TKR <35 SF PCS	Mixed	-0.008 QALY	Life-time	€ 799 548/QALY Lost
van den Houten et al. (2016)	2016	Netherl	Intermittent claudication	Endovascular revascularization (ER)	Markov	−0.07 QALYs	5 years	€ 106 140/QALY Lost
Howard et al. (2017)	2017	UK	Leukaemia	FCM-miniR	Mixed	-0.059 QALYs	Life-time	€ 147 765/QALY Lost
Kievit et al. (2016)	2016	Netherl	Rheumatoid arthritis	Dose optimisation	RCT	−0.02 QALYs	18 months	€ 681 444/QALY Lost
Ladabaum et al. (2020)	2020	UK	Colorectal cancer	Tailored colonoscopy	Decision Model	- 0,0015 QALYs	Lifetime	€193 353/QALYs
Latimer et al. (2013)	2013	UK	Hospital Falls	New Flooring	Mixed	-0.006 QALY	Life-time	€ 198 120/QALY Lost
Mahmoud et al. (2021)	2021	Netherl	Ulcerative colitis	Withdrawal of anti-tumour necrosis factor alpha (TNF)	Markov	-0,04 QALYs	5 years	€ 318 434,85/QALY
Manca et al. (2006)	2006	UK	Neck pain	Brief physiotherapy intervention	RCT	-0.0010 QALY	12 months	€ 116 310/QALY Lost
Navarro et al. (2020)	2020	Spain	Rheumatoid arthritis (RA)	tofacitinib-containing treatment sequences	RCT	− 0.092 QALY	Lifetime	€440 918/QALY
O’Day et al. (2016)	2016	US	Heart Failure	I-mIBG imaging	Decision Model	-0.001 QALYs	2 years	€ 5 044 460/QALY Lost
Oddershed et al. (2016)	2016	UK	HIV	Protease inhibitor	Mixed	-0.0227 QALYs	3 years	€ 379 295/QALY Lost
Okeke et al. (2021)	2021	UK	Missed miscarriage	mifepristone and misoprostol (MifeMiso)	RCT	- 0,04% QALYs	21 days	€425 080/QALY
Shapiro et al. (2017)	2017	US	Breast Cancer	ZA every 3 months	Markov	-0.01 QALYs	2 years	€ 322 672/QALY Lost
Stoecker et al. (2013)	2013	US	Pneumococcal diseases (vaccination)	2+1 pneumococcal vaccine	Prob. Model	-0.005 QALYs	Life-time	€ 285 351/QALY
Thoma et al. (2014)	2014	Canada	Breast Mammaplasty	Vertical Scar Reduction	RCT	−0.01 QALY	1 year	€783 556/QALY Lost
Udkoff and Eichenfield. (2017)	2017	US	Psoriasis	Ixekizumab every 4 weeks	Markov	-0.006 QALYs	5 years	€ 3 138 538/QALY Lost
Wagmiller et al. (2006)	2006	US	Prostate cancer	Individualized schedule	Model	-0.005 QALY	5 years	€ 782 954/QALY Lost
Wailoo et al. (2008)	2008	US	Rheumatoid arthritis	Anakinra	Decision Model	-0.2 QALYs	Life-time	€ 231 773/QALY Lost
Wong et al. (2015)	2015	China	Transitional care	Home visit	RCT	−0.0002 QALYs	28 days	€ 1 175 700/QALY Lost

In total, 78% of the studies evaluated had high or very high quality, bias and transferability scores. Over 90% of the studies included in this review clearly stated their objectives and population characteristics. Only 28 of the economic analyses were carried out from the societal perspective and this typically meant an estimation of productivity costs in terms of absenteeism from work and cost of caregiving. The costs estimated rarely included out of pocket payments or private health insurance payments that can be important in some countries despite universal coverage and social health insurance. Generalisability of the results to other settings were discussed in 73% of papers and ethical and distributional issues were only addressed in two-thirds of papers. For 25% papers no sponsorship information was communicated.

The expanded search to conference abstracts, posters, published protocols and ClinicalTrials.gov registry entries for equivalence or non-inferiority trials, a total of 48 records were identified for in depth investigation and the first authors were contacted to investigate if an economic evaluation had been published. Only 21 replies were received and of these just four studies had a publication available, which were not d-CE.

## Discussion

This review aimed to summarise the existing economic studies of decrementally cost-effective technologies published since 2005. Given its international nature and the variety of effectiveness endpoints, no threshold was used to characterise whether costs savings associated with a loss of health were acceptable or not. However, it is under debate whether or not the willingness to pay (WTP) value would be the same as the willingness to accept value. The societal point of view indicates that WTA is usually higher than WTP, potentially with double the cost difference for one QALY lost than the WTP for one QALY gained (Kievit et al., 2016). The net monetary benefit approach has been advocated, however it still requires a decision on the acceptable loss of efficacy for the non-inferiority condition to be met, as well as scenarios on the decision maker’s willingness to accept thresholds (Xie et al., 2019).

The 54 studies which used QALYs as the measure of outcome reported a wide range of d-CERs, from an unacceptable €151 to a high € 5,044,460 saved per QALY lost, with a fair share of them reporting results above €100,000 saved per QALY lost. However, the C-E results of the studies cannot be directly compared due to methodological differences such as the different economic perspectives, different discount rates and different health systems.

In the Netherlands, the WTA is considered to be € 80,000 saved per QALY lost, although this value has not been officially stated and we had applied this threshold value in our review, we would have excluded more than 30% of the papers. A previous systematic review on d-CE HTs, conducted over the time period 2002–2007, identified just eight d-CE interventions (Nelson et al., 2009).

Besides the growth of economic evaluations published in the recent years and the conservative approach of the above-mentioned study only d-CE interventions being at least $100,000 cost saving for each QALY lost were included. However, there could be other reasons for the higher number of studies found in our review. For example, 46 out of 66 RCT-based economic evaluations were based on non-inferiority or equivalence clinical trials and non-inferiority clinical trials are being performed with a greater frequency every year (Murthy et al., 2012). These trials are usually undertaken to test the hypothesis that the new technology will provide better safety at the cost of an acceptable reduction in efficacy. The addition of an economic analysis using QALYs as outcomes in that situation is highly relevant because 1) the new technology can be cost reducing and 2) both safety and efficacy are covered by the generic health related quality of life measure. Moreover, the time period covered by our review included the austerity measures on healthcare spending caused by the global financial crisis which has been a key driver to decision making based on maximising collective health benefits while controlling costs. Curbing overtreatment and rational prescribing is another key topic in healthcare and nearly half of the 107 days-CE HTs we found were dose reduction/de-escalation interventions (OECD and European Union, 2018).

Next steps of research would involve investigating the opportunity cost generated by implementing d-CE HTs in national settings to identify how to displace the financial savings to maximise population’s health outcomes. For example, using results from the PIVOT trial, it is has been estimated that switching 45,000 HIV patients in the UK from triple antiretroviral therapy to the clinically non-inferior protease inhibitors monotherapy (until viral load rebound) would lead to cost savings that could be used to generate 22,354 QALYs elsewhere, including 1,486 lives prolonged and 6,735 life-years gained (Oddershede et al., 2016). The potentially collective health gains under limited resources have also been described in a model by Arbel et al., which has been used by the German health system when comparing alternative interventions under a pre-specified budget constraint (Arbel and Greenberg, 2016) ^.^ A less expensive and less effective therapy might add more QALYs in a target population when there are budget constraints (Birch and Gafni, 2004). However, cost saving is rarely the primary reason for choosing a particular treatment strategy. In case of HIV, for example, the WHO rejects the provision of cheaper and less effective treatments in any situation, to avoid the establishment of a double standard of care. It can be argued that it should be mandatory for health professionals to provide the best available option to their patients, but from a broader societal perspective, decision makers may claim that is more important to achieve equity in the supply of medical innovations (Persad and Emanuel, 2017). Since 2010, in OECD countries, the expenditure on health has remained relatively flat within a global context of budget constraint. Policy recommendations for implementing slightly less effective medical interventions, but at significantly lower cost, might represent a more effective use of resources to provide additional health gains to the population (Kent et al., 2004) (OECD and European Union, 2018).

Having identified these 107 days-CE HTs, the question remains for policy makers on which of these could be implemented. Interventions that are highly d-CE for pathologies with a significant burden of disease would probably be most pertinent for investigation by HTA agencies and medical associations. Overcoming the reticence of stakeholders to look into the south-west quadrant of the C-E plan and find consensus for a WTA threshold is a context sensitive issue.

One HT found by this systematic review was based on a RCT that demonstrated that for patients with active rheumatoid arthritis, having failed conventional synthetic disease-modifying anti-rheumatic drugs (csDMARD) mono-therapy, triple therapy was non-inferior to the biological disease-modifying anti-rheumatic drugs (bDMARDs) with Methotrexate. The economic analysis estimated an average reduction in QALY of -0.017 and cost savings of $977,805 per QALY lost, mainly attributable to the lower drug costs of csDMARDS (O’Dell et al., 2013) (Bansback et al., 2017). In terms of implementation in Europe, there are different eligibility criteria for reimbursement of bDMARDs depending on the country. For example, in France, where bDMARDs are up to 30 times more expensive than csDMARDs, the eligibility criteria for bDMARD reimbursement do not require minimal disease duration nor that a certain number of csDMARDs fail prior to prescribing a biologic therapy. The percentage of French patients treated with a combination of three csDMARDs was less than 1% in the ESPOIR cohort of 2018. In the UK, where NICE recommends biologics for patients with RA only if the disease activity is severe and has not responded to treatment with a combination of csDMARDs, the National Clinical Audit for RA indicated that at least 46% of English patients received a combination of csDMARDs at some point (HAS, 2019) (Firth et al., 2016). The launch of biosimilar bDMARDs can further affect prescribing habits: the sales of biosimilar Etanercept (Benepali^®^) increased by 172% in France from 2017 to 2018 (Medic’AM, 2018). However, biosimilars are still relatively expensive compared with csDMARDs and thus triple therapy remains the least costly option in people failing csDMARD monotherapy. Since prescribers do not always follow the HTA guidelines, the question of how to motivate them to do so should be addressed. In addition to reimbursement policy, incentives such as novel payment models to encourage use of a less expensive but much cheaper technologies compared to usual care may be necessary (Hutton et al., 2014).

Despite the increased number of d-CE papers found, it is possible that some studies are not published due to the results being unable to demonstrate non-inferiority, equivalence or in the case of superiority trials, health gains, despite the possibility that an economic evaluation may have unearthed d-CE interventions in some of these cases. A technology that is proven non-inferior (ie possibly inferior but within an acceptable margin for clinical outcome) cannot expect a price premium and will usually be launched at a discounted price (10–15% for example) relative to the comparator. In that sense, policy makers and payers have already answered the question of the equivalence margin for costs, although probably did not consider the joint distribution of costs and effects whether they be clinical outcomes or QALYs. Innovative new frameworks may need to be developed to help policy decisions (Xie et al., 2019).

The comparability of study results was limited by the heterogeneity of endpoints in studies that did not use QALYs, and by the lack of standardization in the selection of non-inferiority margins for clinical trials (Waliszewski et al., 2020). We did not address the ethical process of ensuring that disinvestment decisions are acceptable by the population at large (Pace et al., 2020).

## Conclusion

This systematic review has revealed a growth in recent years in the number of economic evaluations of d-CE HTs and identified 107 HTs that are d-CE compared to usual care. Some of these HTs, that represent a potentially large cost saving for a small loss in efficacy, can be examined by decision-makers for uptake in different setting. Economic and policy tools are needed to facilitate the adoption of a decrementally cost-effective health technology in different settings since this should contribute towards the maximisation of population health outcomes.

## Data Availability

The original contributions presented in the study are included in the article/Supplementary Material, further inquiries can be directed to the corresponding author.
