# Supplementary material for: Decrementally cost-effective health technologies in non-inferiority studies: A systematic review
Source: Front Pharmacol. 2022 Dec 5;13:1025326. doi: 10.3389/fphar.2022.1025326 (PMC9760952; doi:10.3389/fphar.2022.1025326)
Supplement: Supplementary file 2 [file Table2.DOCX]

# APPENDIX 1 Full search strategies used for all databases

1. A) PubMed search string

((((("Cost-Benefit Analysis"[MeSH] OR "*economic*" OR "Cost Control"[MeSH] OR "Costs and Cost Analysis"[MeSH] OR"*Cost*" OR "*saving*" OR "*save*" OR "*QALY*" OR "*resource utilisation*" OR "*resource utilization*" OR "*economic*" OR "*expenditure*" OR "*fees*" OR "*charges*" OR "*budget*" OR "*fiscal*" OR "*financ*" OR "*fund*" OR "*price*" OR "*pricing*" ) AND ("*Non inferior*" OR "*Noninferior*" OR "*non-inferior*" OR "*Decremental*" OR "*De escalation trial*" OR "*De-escalation trial*" OR "*disinvest*" OR "*equivalence*" OR "*less effective*"))))) AND ("2005/05/01"[Date - Publication] : "2021/10/04"[Date - Publication])

1. B) ClinicalTrials.gov search string

(Cost-Benefit Analysis OR economic impact OR economic analysis OR cost effective OR cost effectiveness OR Costs and Cost Analysis) AND (Non inferior OR non-inferior OR noninferior OR De escalation trial OR disinvest)

1. C) TUFTS search string

Decremental cost-effective

1. D) EuroCT search string

(Cost-Effective* OR cost effective* OR cost-benefit analysis OR economic* OR Costs and Cost Analysis) AND (non-inferior OR noninferior OR non inferior OR disinvest OR decremental)

1. E) EbscoHost search string

(“Cost-Benefit Analysis”[MeSH] OR “economic impact” OR “economic analysis” OR “Cost Control”[MeSH] OR “Costs and Cost Analysis”[MeSH]) AND (“Resource Allocation”[MeSH] OR “Non inferior*” OR “Noninferior*” OR “non-inferior*” OR “Decremental* cost effective*” OR “Decremental* cost-effective*” OR “De escalation trial*” OR “De-escalation trial*” OR “disinvest”)

1. F) CRD York search string

Cost-Benefit Analysis AND non inferior* trial

1. G) ISRCTN search string

(Non inferior OR non inferiority) AND (cost effective OR cost effectiveness)

1. H) EMBASE search string

('cost effectiveness analysis'/exp OR 'cost effectiveness analysis' OR 'cost benefit analysis'/exp OR 'cost benefit analysis' OR 'cost utility analysis'/exp OR 'cost utility analysis' OR 'cost minimization analysis'/exp OR 'cost minimization analysis' OR 'health economics'/exp OR 'health economics' OR 'economic evaluation'/exp OR 'economic evaluation' OR 'incremental cost effectiveness ratio'/exp OR 'incremental cost effectiveness ratio') AND ('decremental' OR 'equivalence trial'/exp OR 'equivalence trial' OR 'non-inferiority trial'/exp OR 'non-inferiority trial' OR 'disinvest' OR 'less effective') AND [2005-2021]/py AND [english]/lim AND [humans]/lim
