# Supplementary material for: Decrementally cost-effective health technologies in non-inferiority studies: A systematic review
Source: Front Pharmacol. 2022 Dec 5;13:1025326. doi: 10.3389/fphar.2022.1025326 (PMC9760952; doi:10.3389/fphar.2022.1025326)
Supplement: Supplementary file 4 [file DataSheet2.docx]

|  | **YES** | **Some problems** | **NO** | **Not applicable** |
| --- | --- | --- | --- | --- |
| **Quality questions** |  |  |  |  |
| 1. Is the study population well described? |  |  |  |  |
| 2. Are competing alternatives clearly described? |  |  |  |  |
| 3. Is a well-defined research question posed in answerable form? |  |  |  |  |
| 4. Is the economic study design appropriate in order to include relevant costs and consequences? |  |  |  |  |
| 5. Is the chosen time horizon appropriate in order to include relevant costs and consequences? |  |  |  |  |
| 6. Is the actual perspective chosen appropriate? |  |  |  |  |
| 7. Are all important and relevant costs for each alternative identified, measured and valued appropriately? |  |  |  |  |
| 8. Are all important and relevant outcomes for each alternative identified, measured and valued appropriately? |  |  |  |  |
| 9. Is an incremental analysis of costs and outcomes of alternatives performed? |  |  |  |  |
| 10. Are all future costs and outcomes discounted appropriately? |  |  |  |  |
| 11. Are all important variables, whose values are uncertain, appropriately subjected to sensitivity? |  |  |  |  |
| 12. Do the conclusions follow from the data reported? |  |  |  |  |
| 13. Does the article indicate that there is no potential conflict of interest of study researcher(s)? |  |  |  |  |
| 14. Are ethical and distributional issues discussed appropriately? |  |  |  |  |
| **Transferability questions** |  |  |  |  |
| 15. Does the article provide sufficient detail about the study sample(s)? |  |  |  |  |
| 16. Are quantitative and/or descriptive analysis conducted to explore variability from place to place? |  |  |  |  |
| 17. Does the study discuss generalisability of their results? |  |  |  |  |
| **Bias questions** |  |  |  |  |
| 18. Inefficient comparator bias. Was the best alternative chosen as comparator? Was current practice chosen as a comparator? |  |  |  |  |
| 19. Sponsor bias. Have sponsorships been disclosed? Is the study protocol freely accessible? |  |  |  |  |
| 20. Reporting and dissemination bias. Has the study/trial been listed in a trial register? Have all results been reported according to the study protocol? |  |  |  |  |
| 21. Bias related to structure. Is the model structure in line with coherent theory? Do treatment pathways reflect the nature of disease?* |  |  |  |  |
| 22. Bias related to data. Are the methods of data identification transparent? Are probabilities, for example, based on natural history data and identified accurately?* |  |  |  |  |
|  |  |  |  |  |
| **Model-specific aspects of bias in economic evaluation* |  |  |  |  |
